# Photocatalytic Degradation of Tetracycline by ZnO/γ-Fe_2_O_3_ Paramagnetic Nanocomposite Material

**DOI:** 10.3390/nano10081458

**Published:** 2020-07-25

**Authors:** Paola Semeraro, Simona Bettini, Shadi Sawalha, Sudipto Pal, Antonio Licciulli, Fabio Marzo, Nicola Lovergine, Ludovico Valli, Gabriele Giancane

**Affiliations:** 1 Department of Biological and Environmental Sciences and Technologies, University of Salento, Via per Monteroni, 73100 Lecce, Italy; paola.semeraro@unisalento.it; 2 Consorzio Interuniversitario Nazionale per la Scienza e Tecnologia dei Materiali, INSTM, Via G. Giusti 9, 50121 Firenze, Italy; simona.bettini@unisalento.it (S.B.); gabriele.giancane@unisalento.it (G.G.); 3 Department of Engineering of Innovation, University of Salento, Via per Monteroni, 73100 Lecce, Italy; shadi.sawalha@unisalento.it (S.S.); sudipto.pal@unisalento.it (S.P.); antonio.licciulli@unisalento.it (A.L.); fabio.marzo@unisalento.it (F.M.); nicola.lovergine@unisalento.it (N.L.); 4 Department of Chemical Engineering, An-Najah National University, P.O. Box 7, Nablus, Palestine; 5 Department of Cultural Heritage, University of Salento, Via D. Birago, 64, 73100 Lecce, Italy

**Keywords:** tetracycline, photocatalytic degradation, zinc oxide, iron oxide, nanocomposite catalyst

## Abstract

In recent years, the presence of numerous xenobiotic substances, such as antibiotics, has been detected in water environments. They can be considered as environmental contaminants, even if their effect on human health has yet to be totally understood. Several approaches have been studied for the removal of these kinds of pollutants. Among these compounds, tetracycline (TC), a broad-spectrum antibiotic, is one of the most commonly found in water due to its widespread use. In the context of reducing the presence of TC in aqueous solution, in this contribution, a composite catalyst based on zinc oxide (ZnO) and iron oxide (γ-Fe_2_O_3_) was developed and its photocatalytic properties were investigated. The catalytic materials were synthesized by a microwave-assisted aqueous solution method and characterized by Field Emission Scanning Electron Microscope (FESEM), X-Ray Fluorescence (XRF) and Brunauer−Emmett−Teller (BET) analysis. The TC concentration was evaluated by spectrophotometer measurements at specific time intervals. The performed photocatalytic experiments clearly demonstrated that the ZnO/γ-Fe_2_O_3_ composite catalyst presents significant photocatalytic activity, indeed a TC degradation efficiency of 88.52% was registered after 150 min. The presence of iron oxide in the structure of the catalyst enhances both the surface area and the pore volume, facilitating the adsorption of the analyte on the surface of nanostructures, a fundamental phase to optimize a photodegradation process. Moreover, ZnO was found to play the key role in the photocatalytic process assisted by γ-Fe_2_O_3_ which enhanced the TC degradation efficiency by 20%.

## 1. Introduction

Tetracyclines (TC) are a class of broad-spectrum antibiotics widely employed to treat various human and animal bacterial infections [1]. Due to their incomplete adsorption and partial metabolization by humans and animals, more than 70% of the TC used for pharmacological treatment are excreted by feces and urine [2] in zootechnical liquid waste and in municipal wastewaters. The presence of tetracyclines in environmental water induces several effects on soil microbial activity and aquatic photosynthetic organisms, causing an increase in antibiotic-resistant bacteria [3] and significant human health problems [4]. Generally, contamination of water bodies, such as lakes, rivers, streams, oceans, aquifers or ponds, caused by anthropogenic activity, has a relevant impact on the ecosystem and is considered a worldwide cause for alarm [5]. Thus, in the last years, several physical, chemical and biological methods have been developed to remove pollutants and dangerous compounds from water [6,7]. However, traditional biological wastewater treatments are not efficient enough to remove tetracycline due to its low biodegradability [8] and indeed this antibiotic has been found in natural waters [9].

Therefore, the design and development of new technological approaches able to remove tetracyclines from wastewater before their discharge into aquatic environments are required. Among the various wastewater treatments, which include biodegradation, adsorption, electrochemical degradation and ozonation [10,11,12,13], new methods based on advanced oxidation processes (AOPs) have been recently developed [9]. AOPs involve the use of photocatalytic systems, such as zinc oxide (ZnO) or titanium dioxide (TiO_2_) nanoparticles, that produce hydroxyl radicals (∙OH) in situ under electromagnetic radiation [14,15]. These radicals trigger the oxidation of contaminants and convert them into less harmful byproducts [9,14]. In addition, AOPs allow mineralization of recalcitrant organic pollutants into green products with a rapid degradation rate under ambient temperature and pressure conditions [16]. ZnO and TiO_2_, the most used semiconductors in oxidation processes, have a band gap energy of about 3.2 eV and consequently, their excitation is limited to the ultraviolet (UV) radiation range [16,17,18,19,20]. However, ZnO nanoparticles are cheaper than TiO_2_ ones and ZnO photon absorption efficiency at room temperature is higher that the light absorption properties of TiO_2_ under the same conditions [17].

Although numerous studies on the removal of tetracyclines from water using ZnO as catalyst have already been reported [21,22,23,24], in this work, we propose the synthesis of a new nanocomposite material based on zinc oxide and paramagnetic iron oxide, in particular an oxide of Fe(III) in gamma phase (γ-Fe_2_O_3_), employed as catalyst to induce TC degradation in aqueous solutions. The obtained nanocomposite catalyst (ZnO/γ-Fe_2_O_3_) has the advantage of being easily separated from water after the photocatalytic process by means of the application of a weak external magnetic field, so the presence of paramagnetic γ-Fe_2_O_3_ nanoparticles in the ZnO/γ-Fe_2_O_3_ allows a very simple recovery of catalyst and its possible reuse in other degradation cycles. Hence, common methods of solid-liquid separation, such as filtration or centrifugation, can be replaced by the use of a magnetic device. Moreover, the presence of iron oxide allows one to obtain a new composite material with higher area surface and pore volume improving its photocatalytic properties and both the metal oxides promote the degradation of tetracyclines. Thus, the presence of paramagnetic iron oxide in the ZnO/γ-Fe_2_O_3_ plays a dual role: it enhances the photocatalytic activity of ZnO-based nanostructures and it allows the photocatalyst to be quickly and efficiently removed from treated solutions.

## 2. Materials and Methods 

### 2.1. Catalyst Preparation

To synthesize flower-like ZnO structures, a microwave assisted aqueous solution method was used. zinc sulfate heptahydrate (ZnSO_4_ × 7H_2_O, 100 mg, Merck, Darmstadt, Germany) were dissolved in 200 mL of deionized water and then an ammonium hydroxide solution (25% NH_3_ in H_2_O, Fluka, Munich, Germany) was slowly added dropwise to the solution under continuous stirring until a pH value of 11 was achieved. The solution was heated by means of a microwave device (800 W) for 15 min resulting in the formation of a white precipitate. Then, the solution was left to cool to room temperature, the precipitate was collected by centrifugation, washed with water several times, and dried in an oven at 60 °C for 10 h. By this microwave-assisted aqueous solution method it is possible to synthesize three-dimensional structures composed of small nanoparticles. Regarding the growth mechanism, it may be assumed that ZnO crystal nuclei are first polarized by the electrical field of microwave radiation and then form nanoparticles arranged in different layers [25].

Iron oxide nanoparticles capped by polyethylene glycol (PEG) were prepared by means of coprecipitation and solvothermic synthesis according to the procedure reported by Bonfrate and coauthors [26]. Chemicals such as hexahydrate ferric chloride, tetrahydrate ferrous chloride, sodium acetate, polyethylene glycol (PEG 8000), diethylene glycol (DEG) and ethylene glycol (EG) (with a volume ratio DEG:EG = 30:10) were purchased from Merck and used without any further purification. This synthesis allows one to obtain nanostructures based on ferric oxide (γ-Fe_2_O_3_) with paramagnetic features [26,27].

For the synthesis of ZnO/γ-Fe_2_O_3_ composite material, 100 mg of the previously prepared iron oxide nanoparticles capped by PEG were mixed with 100 mg of ZnSO_4_ × 7H_2_O and dissolved in 200 mL of deionized water. Then, the same microwave-assisted aqueous solution method described before for the synthesis of flower-like ZnO nanoparticles was applied, but in this case the obtained ZnO/γ-Fe_2_O_3_ were simply collected by means of a neodymium magnet and washed three times in ethanol and then in deionized water. During this synthesis, it can be supposed that the iron oxide grains are placed between the ZnO aggregates and this inhibits the formation of the peculiar 3D flower-shape structures [28].

### 2.2. Catalyst Characterization

X-ray diffraction profiles of the synthetized nanostructures were recorded by a Miniflex diffractometer (Rigaku, Tokyo, Japan) from 20 to 80°. The step was 0.020° and the scan speed 0.25°/min. A CuKα radiation at 30 kV and 100 mA was used as X-rays source.

The morphology of the synthesized structures was analyzed by a Field Emission Scanning Electron Microscope (FESEM) on a Sigma VP microscope (Zeiss, Oberkochen, Germany) equipped with a Gemini electron column.

To determine the stoichiometric ratio of Zn and Fe in the composite catalyst, X-Ray Fluorescence (XRF) spectroscopic measurements were performed by using a M4 TORNADO Micro-XRF spectrometer (Bruker, Billerica, MA, USA).

Brunauer−Emmett−Teller (BET) method was used to determine the specific surface area and porosity of the photocatalytic materials. The analysis was carried out by a Quantachrome NOVA 2200e series surface analyzer (Boynton Beach, FL, USA) using N_2_ adsorption/desorption at 77 K. Before performing the measurements, the samples were degassed under a N_2_ atmosphere for 12 h at 120 °C.

### 2.3. Photocatalysis Experiments

In each experiment, the opportune amounts of ZnO/γ-Fe_2_O_3_ catalyst were added in 20 mL of an aqueous solution of tetracycline (Merck, Darmstadt, Germany) with a concentration of 30 mg/L and the system was maintained under continuous stirring (250 rpm) at room temperature and at pH equal to 6.7. The effect of different nanocomposite catalyst dosage (5, 10, 15 mg) was tested and 10 mg (0.5 mg/mL) of ZnO/γ-Fe_2_O_3_ catalyst was used for the most of measurements carried out. It will be pointed out that this value represents the optimum amount of catalyst for TC photodegradation. The TC degradation efficiency of ZnO flower-like and ZnO/γ-Fe_2_O_3_ catalysts were also compared. The photocatalytic activity of the synthesized materials was evaluated by time-monitoring the TC decomposition under UV-visible light irradiation. Before the illumination, the system was kept in dark for about 15 min to reach the adsorption-desorption equilibrium, then was irradiated by simulated solar light using a Solar S class A (100 mW cm^−2^ light intensity) halogen lamp (LOT-Oriel Italia Srl, Rome, Italy). The illumination experiments were carried out for 150 min because no significant improvements of degradation efficiency were observed at greater times. Every 30 min, an aliquot (2 mL) of TC solution was collected and separated by catalyst to measure the corresponding concentration of TC by an UV-Vis spectrophotometer (Lambda 650 UV/VIS Spectrometer, PerkinElmer, Waltham, MA, USA). The absorbance value at the characteristic band of about 360 nm was considered.

To calculate the TC degradation efficiency (*E*), the following Equation (1) was used:

(1) $$E\;\left(\%\right)=\frac{C_{0}-C_{t}}{C_{0}}\times 100,$$

where *C*_0_ and *C_t_* are the concentrations of tetracycline (mg/L) at the initial and degradation time.

The kinetics of photodegradation reaction of TC were studied by first-order kinetics model expressed in Equation (2), according to the Langmuir–Hinshelwood kinetics model [14]:

(2) $$ln\frac{C_{0}}{C_{t}}=kt,$$

where *k* and *t* are respectively the kinetic rate constant and irradiation time. The *k* value indicates the photocatalytic activity and it was calculated by the linear plot slope.

In order to investigate the main photodegradation mechanism, the production of radicals (∙OH and O_2_^−^) and holes (h^+^) was tracked by adding some radical oxygen species (ROS) scavengers, such as isopropanol (IPA; ∙OH scavenger), L-ascorbic acid (LAA; ∙O_2_^−^ scavenger) and ethylenediamine-tetraacetic acid disodium salt (EDTA-Na_2_; h^+^ scavenger). Each aqueous solution of scavenger with a final concentration of 1 mM was added to TC solution (30 mg/L) in presence of ZnO/γ-Fe_2_O_3_ nanocomposite catalyst. All measurements were performed in triplicate to ensure the reproducibility of the results and the mean values were reported.

To probe the recyclability of the catalyst, consecutive photocatalytic experiments were performed using the same ZnO/γ-Fe_2_O_3_. After the first photocatalytic process, the catalyst was used three times in subsequent TC photodegradation reactions. In each experiment, the catalyst was separated from the solution by a neodymium magnet, washed carefully with deionized water, dried and reused in the next degradation cycle under the same conditions.

## 3. Results

### 3.1. Catalyst Characterization

**Figure 1:**
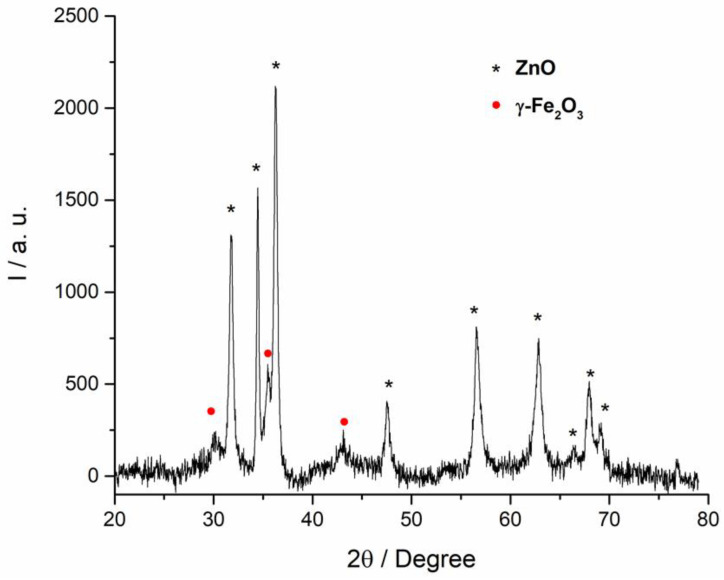
XRD pattern of ZnO/γ-Fe_2_O_3_ nanocomposite system. In the graph the peaks related to the ZnO are labeled with black stars and the peaks imputable to the maghemite are indicated with red circles.

ZnO/γ-Fe_2_O_3_ nanostructures were characterized by means of XRD in order to identify the crystalline phase of the two components. As reported in Figure 1, ZnO shows the typical diffraction pattern of the zinc oxide in the wurtzite form (JPCS card 036-1451) and the iron oxide seems to maintain the original maghemite phase [29]. 

**Figure 2:**
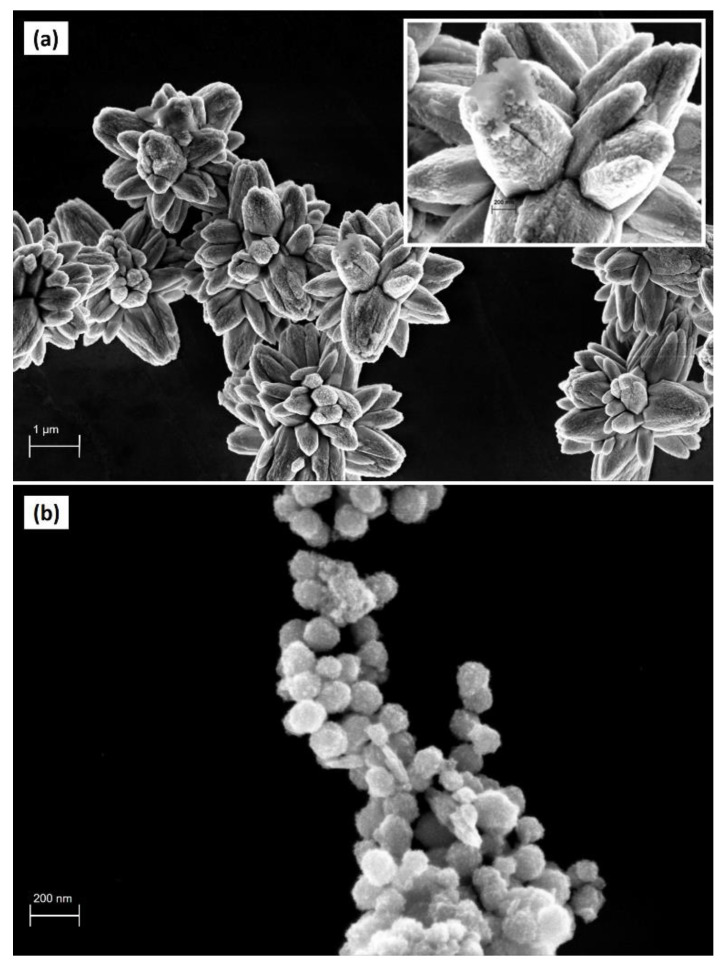
(**a**) FESEM image of ZnO flower-like catalyst; (**b**) FESEM image of ZnO/γ-Fe_2_O_3_ composite catalyst.

FESEM measurements were performed to investigate the morphological details of the synthesized catalysts. In Figure 2a, it is possible to clearly observe a three-dimensional flower shaped ZnO structure with a width of about 2.5–3 μm. Moreover, the magnified FESEM image inserted in Figure 2a highlights that the surface of the flower-shaped structures appears very rough, an interesting feature for a photocatalyst.

However, in the ZnO/γ-Fe_2_O_3_ composite material, the characteristic flower shape is not preserved because of the iron oxide nanoparticles that are located both inside and outside of the ZnO microstructure. FESEM image of ZnO/γ-Fe_2_O_3_, shown in Figure 2b, reveals a surface densely covered by paramagnetic spherical grains with a diameter size of about 80–100 nm, in good agreement with the literature [26].

The weight percentage of Zn and Fe oxides in the structure of the synthetized composite catalyst was measured by XRF analysis. The amount of the oxides in the composite calculated from the XRF spectra (Figure S1) was found to be 40.07 (wt. %) for ZnO and 59.32 (wt. %) for Fe_2_O_3_ as reported in Table S1. The amount of ZnO present in the composite material will be used to calculate the opportune dosage of ZnO/γ-Fe_2_O_3_ required in the catalytic processes.

**Figure 3:**
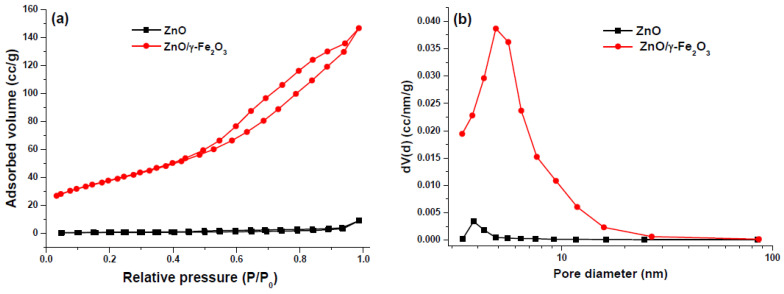
(**a**) N_2_ adsorption−desorption isotherm of ZnO flower-like and ZnO/γ-Fe_2_O_3_ nanocomposite catalysts; (**b**) Pore diameter and pore volume calculated from the desorption branch of isotherm by BJH.

In Figure 3a the N_2_ adsorption−desorption isotherm of ZnO flower-like and ZnO/γ-Fe_2_O_3_ nanocomposite catalysts are reported. The specific surface area was calculated by the multipoint BET method from the N_2_ adsorption branch of the isotherm, the pore diameter and pore volume were calculated from the desorption branch of isotherm using the Barret−Joyner−Halenda (BJH) method (Figure 3b).

The respective values reported in Table 1 emphasize that both the surface area and pore volume of the ZnO/γ-Fe_2_O_3_ nanocomposite are significantly greater than those ones of the ZnO flower-like catalyst. This substantial difference is very probably due to the iron oxide grains that are placed not only on the external surface of composite material, but also between the ZnO particles.

### 3.2. Photocatalysis Experiments

#### 3.2.1. Effect of ZnO/γ-Fe_2_O_3_ Catalyst Dosage

**Figure 4:**
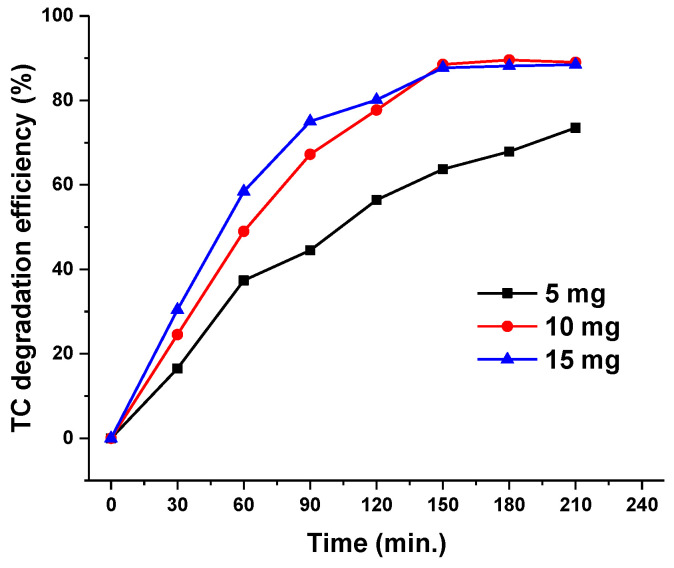
Degradation efficiency of TC (%) vs irradiation time (min) curves of 20 mL of TC solutions (30 mg/L) obtained respectively in presence of 5, 10 and 15 mg of ZnO/γ-Fe_2_O_3_ composite catalyst.

The effect of dosage of ZnO/γ-Fe_2_O_3_ composite material on the TC degradation was investigated by adding 5, 10 and 15 mg of catalyst, respectively, to 20 mL of TC solution (30 mg/L). The results shown in Figure 4 indicate that initially the increase of ZnO/γ-Fe_2_O_3_ dosage enhanced the TC degradation efficiency due to the intensification of the photocatalytic activity. 

When 15 mg of ZnO/γ-Fe_2_O_3_ were added to TC solution, a slight rise in degradation efficiency was initially observed, however at 150 min of irradiation no further increase of TC degradation occurred, probably due to the lack of tetracycline molecules. Consequently, all measurements were performed using 20 mL of TC solutions at concentration of 30 mg/L in which were added 10 mg of ZnO/γ-Fe_2_O_3_ composite catalyst.

#### 3.2.2. Comparison Between ZnO Flower-Like and ZnO/γ-Fe_2_O_3_ Composite Catalysts

**Figure 5:**
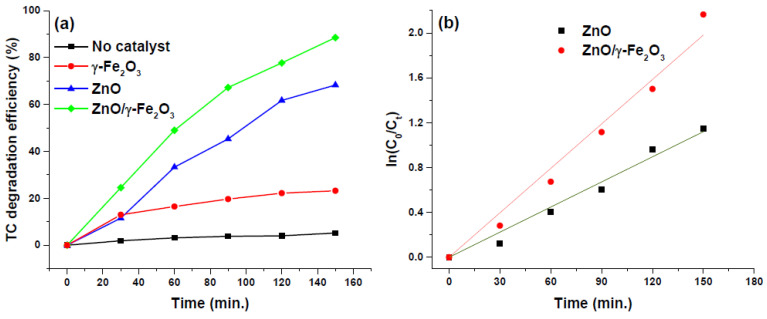
(**a**) Degradation efficiency of TC (%) vs irradiation time (min) curves obtained in the absence of catalyst, and in presence of ZnO flower-like and ZnO/γ-Fe_2_O_3_ composite catalysts; (**b**) First-order fitting plot to study the kinetic of TC photodegradation reaction catalyzed by ZnO flower-like and ZnO/γ-Fe_2_O_3_ composite materials.

In Figure 5a the values of TC degradation efficiency in function of the irradiation time are reported and the photocatalytic activity of ZnO flower-like and ZnO/γ-Fe_2_O_3_ composite catalysts is compared. To perform the comparison between the catalysts, the same quantity of ZnO (0.2 mg/mL) was used considering that the ZnO/γ-Fe_2_O_3_ composite material is made up of about 40% in weight of ZnO, as detected by XRF measurements. In the absence of catalyst no degradation of tetracycline was observed, whereas the γ-Fe_2_O_3_ grains induced a very weak TC degradation. However, in the presence of ZnO flower-like and ZnO/γ-Fe_2_O_3_ composite catalysts a remarkable photocatalytic activity was observed during the light irradiation. After 150 min of irradiation, the TC degradation efficiency values were 68.28% and 88.52% in the presence of ZnO and ZnO/γ-Fe_2_O_3_ catalyst, respectively. This is coherent and in good agreement with ZnO nanorods/magnetite systems used to degrade tetracycline in aqueous solution [30]. With the addition of iron oxide into the composite material, the degradation efficiency of TC was 1.30 times higher than that ZnO flower-like catalyst. The photocatalytic activity enhancement in presence of iron oxide will be considered in the Discussion section.

The photocatalytic decomposition of TC was plotted as first-order fitting (Figure 5b) and the obtained value of kinetic rate constants k for ZnO and ZnO/γ-Fe_2_O_3_ were 7.47 × 10^−3^ min^−1^ and 13.21 × 10^−3^ min^−1^ respectively, as reported in Table 2. The kinetics study of TC photodegradation also confirms the improvement of photocatalytic performance in presence of the γ-Fe_2_O_3_ grains into the structure and on the surface of ZnO with a k value higher by 1.77 times. Moreover, it is commonly accepted that the catalyst morphology affects the photocatalytic properties because the degradation reactions take place at the interface of catalyst where the produced radicals interact with pollutants molecules [31]. Consequently, the large specific surface area of the ZnO/γ-Fe_2_O_3_ makes this composite material more favorable than ZnO for the photocatalytic activity.

## 4. Discussion

As it is well-known [16], photocatalytic phenomena are influenced by both the surface porosity and the energetic levels of the catalyst. When a catalyst is irradiated by light of opportune wavelength, it adsorbs photon energy (hν) equal to its band gap, and the electrons (e^−^) in the valence band (VB) are promoted to the empty conduction band (CB) leaving the positive charged holes in the VB. The holes can react with the water molecules to promote the formation of ∙OH radicals whereas the photogenerated electrons react with dissolved O_2_ to form superoxide species (∙O_2_^−^) and subsequently produce ∙OH [31,32].

From Figure 5a, it is possible to observe that the degradation efficiency of the ZnO/γ-Fe_2_O_3_ can be considered as the algebraic sum of degradation efficiency of ZnO and γ-Fe_2_O_3_ separately. This suggests that the two oxides do not compete or cooperate in the photodegradation process. The predominant photodegradation is due to the zinc oxide, but the γ-Fe_2_O_3_ contributes up to 20% in the total photodegradation efficiency. Furthermore, the paramagnetic features of γ-Fe_2_O_3_ are preserved when the composite nanostructure with ZnO is formed. This is a crucial point since it allows one to quickly separate the nanostructures from water solutions. Thus, iron oxides play a dual role: they improve the photodegradation efficiency and increase the ease of removal of the photocatalyst from aqueous samples.

**Figure 6:**
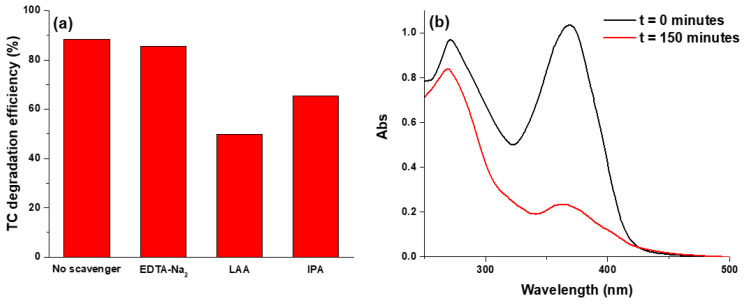
(**a**) Degradation efficiency of TC (%) of ZnO/γ-Fe_2_O_3_ composite material in presence of ROS scavengers after 150 min of light irradiation; (**b**) UV-Vis adsorption spectra of a TC solution (30 mg/L) in presence of ZnO/γ-Fe_2_O_3_ composite material (0.5 mg/mL) before and after 150 min of light irradiation.

Again, to clarify the photodegradation mechanism of tetracycline, radicals and hole scavengers were used. As shown in Figure 6a, the TC photocatalytic degradation in presence of ZnO/γ-Fe_2_O_3_ nanoparticles was considerably inhibited in presence of LAA, moderately reduced by IPA, whereas the addition of EDTA-Na_2_ did not suppress the photocatalytic degradation efficiency. These results suggested that the TC degradation should be mainly influenced from the ∙O_2_^−^ and ∙OH radical oxidative species which can oxidize the TC molecules in other mineralization products. In particular, when IPA is used as ∙OH radical scavenger, it can be supposed that h^+^ in valence band are not used in the degradation process reducing the lifetime of the charge-separated state and, then, increasing the charge recombination rate [33]. This phenomenon reduces, of course, the photodegradation efficiency. On the other side, when h^+^ scavenger is used, the main mechanism of hydrogen peroxide formation, is inhibited even though this process appears not favorable from an energetic point of view [34]. In fact, the VB of ZnO and γ-Fe_2_O_3_ are located at 2.7 and 2.3 eV, respectively. Conduction bands of the zinc oxide and maghemite are reported to be at −0.5 and 0.1 eV, respectively. These evidences exclude the possibility of a charge separation at the ZnO and γ-Fe_2_O_3_ interface, confirming that the two nanostructures cannot cooperate in the photodegradation process and the H_2_O_2_ formation by water oxidation is unlikely.

Finally, as reported in the literature [17], the two absorption bands at about 275 and 360 nm of an aqueous solution of tetracycline can be ascribed to the conjugated double-bond structures with two carbonyl groups and enolic groups, respectively [35]. The tetracycline absorption spectra, in the presence of ZnO/γ-Fe_2_O_3_ catalyst, at the initial time and after 150 min of light irradiation are shown in Figure 6b. After the photocatalysis process an important decrease and a slight hypsochromic shift of the absorption peak at greater wavelengths were observed. These results suggest that under the oxidation process conditions, tetracycline molecules were mineralized and at the same time intermediate products were formed [36,37].

**Figure 7:**
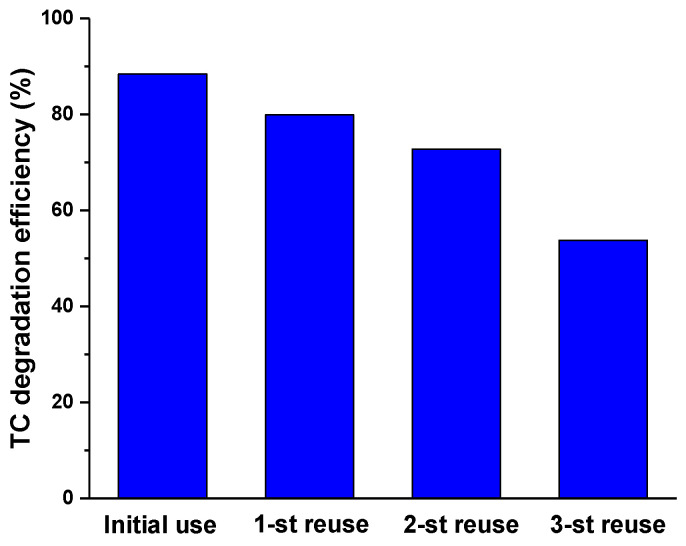
TC degradation efficiency (%) of ZnO/γ-Fe_2_O_3_ composite material cycled for three times after 150 min of light irradiation.

In Figure 7 the TC degradation efficiency (%) in the initial photocatalytic process and in the next three reuse cycles were compared. It is possible to affirm that the ZnO/γ-Fe_2_O_3_ composite material is quite stable during the first two reuse cycles, showing a good recyclability of the catalysts [20,37]. The recyclability of a catalytic material is a very important feature for the feasibility and cheapness of the process studied.

## 5. Conclusions

Tetracycline aqueous solution photodegradation was promoted using a nanostructure formed by ZnO and maghemite under solar irradiation. The presence of iron oxide nanoparticles strongly influences the morphology of the ZnO even though it was highlighted that the crystalline phase of ZnO is preserved (wurtzite form) as well as that of the iron oxide (γ-Fe_2_O_3_) nanostructures. The paramagnetic properties of γ-Fe_2_O_3_ nanoparticles allow one to efficiently remove the photocatalyst from aqueous solutions by simply applying a weak external magnetic field. This substantially reduces the waiting time due to the gravimetric separation of the nanostructures from the aqueous samples proposing the nanocomposite ZnO/γ-Fe_2_O_3_ for application in large scale and continuous water treatment processes. Furthermore, it was demonstrated that the presence of γ-Fe_2_O_3_ increases the porosity and the adsorption properties of the nanostructures if compared to the bare ZnO obtained by the same synthetic protocol. This is an interesting achievement since, as it is known, the analyte adsorption represents the first step of the photodegradation process. Again, it was demonstrated that γ-Fe_2_O_3_ does not affect the ZnO-mediated photodegradation, actually enhancing the catalytic performances of about 20%. In conclusion, the ZnO/γ-Fe_2_O_3_ is able to photodegrade more than the 88% of the water-dissolved TC and, by means of the paramagnetic γ-Fe_2_O_3_ it is possible to quickly remove the photocatalyst and to re-use it, showing its good recyclability features.

## Figures and Tables

**Table 1:** Specific surface area and porosity distribution of ZnO flower-like and ZnO/γ-Fe_2_O_3_ nanocomposite catalysts.

Catalyst	Specific Surface Area (m^2^ g^−1^)	Pore Diameter (nm)	Pore Volume (cc g^−1^)
ZnO	2.193	3.807 ^1^	0.015 ^1^
ZnO/γ-Fe_2_O_3_	133.913	4.859 ^1^	0.210 ^1^

^1^ Calculated from the N_2_ desorption branch of isotherm.

**Table 2:** Kinetic values calculated by first-order kinetics model.

Catalyst	*k* (min^−1^)	*t*_1/2_ (min) ^1^	*R* ^2^
ZnO	0.00747	92.79	0.00747
ZnO/γ-Fe_2_O_3_	0.01321	52.47	0.01321

^1^*t*_1/2_ = (*ln*2)/*k*.

## Supplementary Materials

The following are available online at https://www.mdpi.com/2079-4991/10/8/1458/s1, Figure S1: XRF spectra of the ZnO/γ-Fe_2_O_3_ composite photocatalyst, Table S1: calculated wt. % of the oxides present in the composite photocatalyst.
