# Supplementary material for: Photocatalytic Degradation of Tetracycline by ZnO/γ-Fe2O3 Paramagnetic Nanocomposite Material
Source: Nanomaterials (Basel). 2020 Jul 25;10(8):1458. doi: 10.3390/nano10081458 (PMC7466472; doi:10.3390/nano10081458)
Supplement: Supplementary file 1 [file nanomaterials-10-01458-s001.pdf]

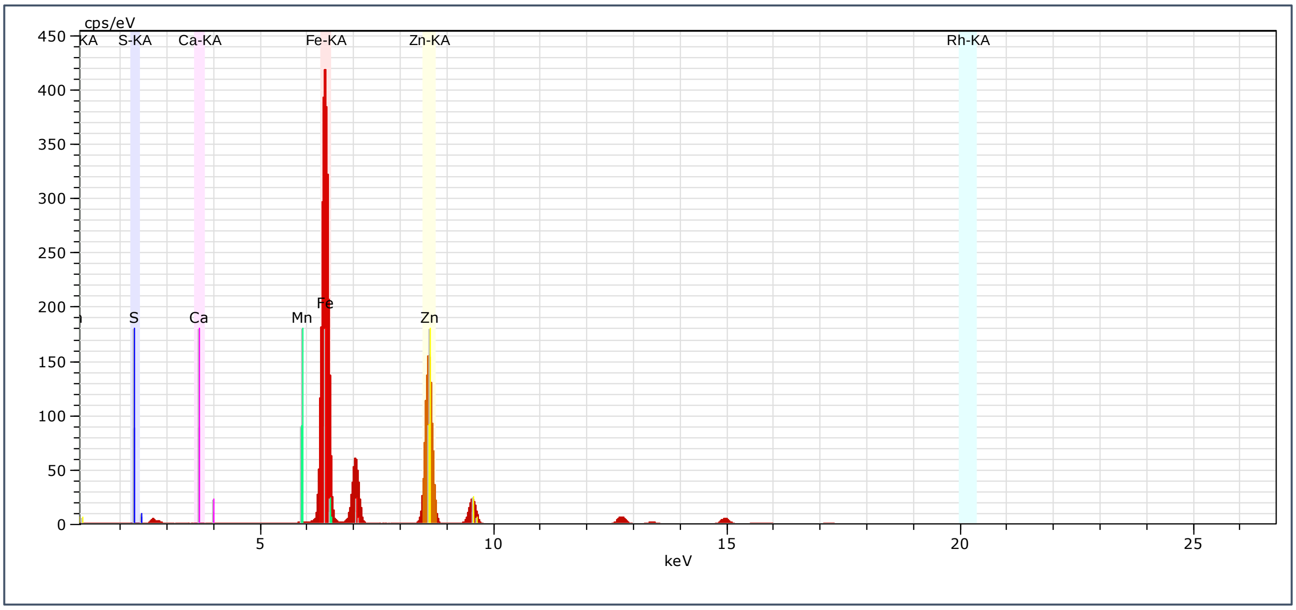

Figure S1. XRF spectra of the ZnO-IO composite photocatalyst. The elements present in the composite material are shown in the spectra according to their individual peaks.

Table S1. Calculated wt% of the oxides present in the composite photocatalyst.

Compounds	Amount (wt%)
Fe_2O_3	59.32
ZnO	40.07
MnO	0.17
CaO	0.04
SO_3	0.40
